# Operative Dentistry

**Published:** 1895-05

**Authors:** 


### Monthly Digest.

### Operative Dentistry.

(*Continued from Page* 201.)

### PREVENTIVE DENTISTRY

Is the subject of a paper by Dr. I. P. Wilson.* The first great preventive measure is oral hygiene, in which it is the duty of every dentist to instruct his patients—especially parents—that they may in turn instruct and properly care for their children, and to do this successfully, the dentist should himself be the example of what he would have his patients be. The writer said: "It is the privilege of every dental practitioner to build up a clean, desirable practice that will not give him discomfort nor endanger his health." This being admitted, it follows that those who fail to appreciate information and advice along these lines, and who persist in the disregard of hygienic laws, asking only for *freedom from pain*, should be dismissed, out of self-respect and regard for health and comfort. A very important point in preventive dentistry is the proper treatment of enamel fissures in immature teeth. The writer advises against any excavation—syringe thoroughly with warm water, dehydrate with alcohol, dry thoroughly and fill with cement, placing the dry powder over the filling and pressing firmly with the finger, forcing the cement into the remotest recesses of the fissures, holding the finger in place to exclude moisture until crystallization has taken place. This will preserve the teeth through the early grade of immaturity, preserving all of the tooth structure, retaining the natural color, and without inflicting pain. "Preventive dentistry should be practiced more by the profession than it is, and our patients will not be slow to appreciate our efforts and remunerate us more willingly for preventive treatment than for more taxing services."

* Dental Digest, February, 1895.

### PYORRHŒA ALVEOLARIS—ALVEOLAR ABSCESSES ON TEETH CONTAINING LIVING PULPS.

A short paper on this subject, citing a typical case, by Prof. W. D. Miller, was read at the American Dental Society of Europe.* He said: "Abscesses of this kind are now put down as the result of a deposit of uric acid, or urates, in the pericementum, in consequence of a general gouty diathesis, and is considered as evidence in support of the view that pyorrhœa alveolaris must have a similar origin."

In the discussion of the subject, DR. HUGENSCHMIDT said that when a patient, showing buccal manifestations of this diathesis, has no knowledge of gout in the family, skillful questioning will generally elicit the presence of rheumatism, migraine, asthma, eczema, or other manifestations of the arthritic diathesis.

DR. BRYAN cited a case found in his own mouth, and said that Prof. Black had recently described this class of cases, which he classifies as a distinct form of pyorrhœa.

DR. MITCHELL also cited a case similar to that described by Prof. Miller. The swelling was about the size of a pea. There was no pocket, and nothing else abnormal visible. The pulp was alive.

The same subject was discussed by the Academy of Stomatology (Phila.).† DR. BURCHARD described a case in which the swelling was located near the apices of the roots of the superior lateral and central incisors, with marked fluctuation at the summits of the swellings and some turgescence of surroundings. The dental ligaments were intact at the gingival margin, with no evidence of inflammation for half an inch above. The swellings disappeared with the administration of tartarlithine, but reappeared exaggerated, coincident with an attack of acute muscular rheumatism.

DR. KIRK cited cases in his own mouth, and said: "The best way to know a thing is to know it by personal experience." The pain, he said, was such as "any one who has had an alveolar abscess knows a great deal about." He is very sure there is not

* Dental Digest, February, 1895.

† International Dental Journal, March, 1895.

a dead pulp in his own mouth. He has also a duplicate case in the mouth of a lady patient. He believes there will be a record of pyorrhœa following this latter acute inflammatory outbreak, and that in these cases we have almost certainly the original lesion which induces the pyorrhœal condition—or the origin of one variety of it.

Dr. DEAN has a similar case, the tooth responding to all tests for vitality.

Dr. GASKILL had had a case with fistula between the first molar and bicuspid. Being unable to determine from which tooth the trouble arose, he drilled into the bicuspid and found a vital pulp, as also in the molar. The fistula was healed by systemic treatment. The same condition presented later, on the other side of the mouth.

DRS. CURRY, REGISTER, ROBERTS and LOUIS JACK cited other cases of abscesses on sound, live teeth. D. JACK said that he had seen so many cases that he is confident they exist more frequently than is supposed.

DR. TRUMAN is skeptical on the point, and questions if proper discrimination is made between vitality of the roots. The pulp in one root may be entirely dead, and in the other still alive, and in that case abscesses would occur from the dead pulp in one root. He said: "I have no belief that abscesses could occur near the apex of the tooth, and the tooth, or that root, retain its vitality." In the cases cited above, however, a number of the live teeth having abscesses were single-rooted teeth, *viz:* Dr. Miller's, a left superior central incisor; Dr. Bryan, a lower incisor; Dr. Burchard (Dr. Gilliam's patient), a superior lateral and central incisor; Dr. Kirk, an upper cuspid; Dr. Deane, a lower right central; Dr. Register, a lateral incisor.

DR. A. V. ELLIOTT, Florence, Italy, read a paper on "Pyorrhœa Alveolaris," before the American Dental Society of Europe.* He said that without going into the hair-splitting questions which are current to-day, practical experience had taught him that there can be no hard-and-fast rule applied to this disease; its most striking feature is its contrariness; the *Eureka* of

* Dental Digest, February, 1895.

yesterday becomes the discouragement of to-day. Though the pathology of the disease had not received very close study previous to the days of Riggs, he has found evidence in the skulls of the most ancient inhabitants of earth, of the ravages of this disease from the earliest days of man on the earth, leading him to the conclusion that "a given set of conditions invariably produce a given result," and that pyorrhœa alveolaris is but "the result of a tendency in the original constitution, acted upon and developed by favorable circumstances." A bad way of living induces bad blood and impure secretions. Nature will not suffer violence without revenging herself sooner or later. He mentions among the favoring conditions in those predisposed to this disease, *first*, a lack of exercise of the teeth, and, *second*, improper cleansing of the teeth. There is nothing new in the treatment indicated in the paper—the usual removal of deposits, washing out of pockets, disinfecting mouth wash, a stimulant application to the gums, and gum massage; finally, exercise by "chewing gum or anything else except tobacco, in which case the remedy would be worse than the disease."

To a similar treatment, Dr. Du Bouchet (Paris) adds: "For those obstinate cases considered as a manifestation of the uric acid diathesis, a regime of *sweet cider*," by the use of which he has obtained a number of vastly improved cases, as well as some apparently cured—the rationale of the treatment being that "through the systemic action of the malic acid, uric acid is transformed into hippuric acid, which has not the inconveniences of the former."

### CANCER OF THE TONGUE.

Dr. E. W. Stevens (M. D.) read a paper on this subject before the Academy of Stomatology (Phila.).*

He said that while sarcoma of the tongue is rarely seen, epithelial cancer is more frequently met with in the tongue than in any other organ, and is much more frequent in men than in women. Leucoma and other pre-cancerous conditions or affections of the tongue were described, and the danger of the appli-

* International Dental Journal and Dental Cosmos, March, 1895.

cation of caustics emphasized as being the most certain means of transforming a simple into a cancerous ulcer. He pointed out the importance of diagnosis at an early stage, and especially of the recognition of the pre-cancerous stage and the diseases with which cancer of the tongue is most likely to be confounded—the dyspeptic ulcer, the tuberculous ulcer, etc. (In the discussion he said: "The members of the dental profession have the best opportunity to observe these various conditions which I have endeavored to describe. There is a good deal to be learned, and it is only by observation that we can finally procure much information about it.")

The hope of surgery for the future, in regard to lingual cancer, lies in prevention rather than in cure. As pre-cancerous conditions become more easily recognized by those who have the earliest opportunity of seeing them, and as the best methods of treatment in the earliest stages become familiar, so may we expect to see a decrease in the number of cases of lingual cancer. For the latter there is but one method of treatment, *viz:* Surgical operation. This may effect a cure in a small number of cases, while it usually prolongs life, and nearly always affords great relief.

In the discussion, the difficulty of distinguishing between benign and malignant tumors, and the undeterminate nature of both clinical and microscopic diagnosis was dwelt upon, the microbic origin of cancer being considered as not proven.

In reply to a question, Dr. STEVENS gave the formula of the wash preferred by him: ten grains of chromic acid to the ounce of water, which seems to ease pain and act beneficially.

Chromic acid is a superficial caustic, while nitrate of silver penetrates very deeply and acts as an irritant.

DR. ROBERT W. GREENLEAF (M. D.) read a paper before the Boston Academy of Dental Science* on "The Relation of Modern Therapeutics to the Practice of Dentistry." Contrasting historical, empiric and modern therapeutics, the former was defined as a "make-believe" stage, and the empiric an intermediate phase which has given much of value, while the therapeutist of

* International Dental Journal, March, 1895.

to-day classifies his weapons in the battle against disease with some approach to a scientific system. Drugs are only one of his many resources, used in conjunction with the remedial agencies of heat and cold; of hygienic measures—as food and rest; electricity, massage, surgical appliances and operations, hypnotism, used for centuries, yet waiting till to-day to be taken out of the bondage of ignorance and viewed calmly in the light of science—these are all collateral fields of modern practical medicine.

After defining the various divisions, classes and sub-classes of medicinal agents now in use, the essayist dwelt more at length upon the classes most essential to the dentist, placing him, in the use of them, upon the same basis as other specialists—aurists and oculists, *viz:* the ability to recognize that a need exists—then to advise proper counsel from the specialist competent to give it. He said: "To obtain such an ability it is necessary to study the fundamental principles of medicine, as one is not likely to see things of which he has no knowledge. With it, however, the medically-trained dentist will find many cases in which his patients are needlessly suffering because they are not given medical aid. . . . Moreover, with such knowledge, the dentist will find that he is far better equipped for understanding the problems of his own specialty." Of the agents more immediately required by the dentist, Dr. Greenleaf spoke of the *antiseptics* as worthy of most careful study, for the control of inflammatory processes so intimately connected with the presence of micro-organisms.

Of the *anæsthetics* he said: "It seems to me that every dentist should understand the use and dangers of anæsthetics, also their indications and contra-indications; then, if he chooses, he may use them safely. If he will not learn these matters he must be regarded as criminal to dare to use such weapons."

Of other groups, as antipyretics, expectorants, sialagogues, emetics, cathartics, anthelmintics, he said, . . . "each contains some drug, the need of which may be clearly indicated in certain patients under your care, who, but for your friendly counsel, might go unrelieved for years, or suffer serious illness which might have been prevented."

DR. FAUGHT'S paper on "The Systemic Treatment of Dental Diseases" (see REGISTER, Jan. issue, p. 48), was discussed at some length by the New Jersey State Dental Society.* The question, "How far does it come within the province of the dentist to practice in this direction?" was raised by Dr. Elliott. He said that, holding the degree of M. D. himself, he had enlarged his practice perhaps further than is usual among dentists, and had been taken to task for having practiced medicine without a license under the medical law. What is the limit of legitimate practice? This is the question that confronts us, and it must be decided.

DR. FAUGHT, in closing the discussion, said: "You are only held responsible for the exercise of due care in the practice of your profession. . . . The line of demarkation and the line of protection lie in your own personality; go to work and get the knowledge and acquire the ability to take care of yourselves in the use of these remedies. . . . The best men in the medical profession will take you by the hand and help you in diagnosing the case. . . . Instead of jealousy, I have had reason to thank the members of the medical profession in helping me out in these matters."

FRACTURES OF THE MAXILLA.

The question of originality and priority in certain methods of treating simple fractures of the lower jaw is discussed† in an interchange of spicy letters between Drs. E. H. Angle and T. L. Gilmer. Evidence in the shape of dates and references is furnished by both parties, with the result that the splint in question is found to be not the invention of either Dr. Angle or Dr. Gilmer, but belongs to Mr. Tomes, being fully described in Heath's "The Injuries and Diseases of the Jaws," as pointed out by Dr. Gilmer himself.

CLEFT PALATE.

DR. C. S. CASE presented to the Chicago Dental Society‡ four

* Dental Cosmos, March, 1895.
†Dental Review, February 15, 1895.
‡Dental Review, February 15, 1895.

patients who testified to the benefits derived from operations for cleft palate.

The subject and methods of operating were discussed at length by Drs. T. W. Brophy, G. V. Black, A. B. Freeman, T. L. Gilmer, Dr. Case and others.

### PULP CAPPING.

Dr. C. R. Taylor* suggests capping pulps with cement applied on a suitably-sized piece of clean writing paper, the paper acting as a convenient carrier, and being also a splendid non-conductor.

### PULP DEVITALIZATION.

Dr. A. G. Johnson, in a paper entitled "Arsenic," read before the Odontographic Society,† traces the history of the use of arsenic in dentistry, in different forms and combinations, giving the following formula:

℞
Arsenious Acid gr. xx.
Hydrochlorate of cocaine gr. xxv.
Lanolin q. s. ft. parte as the most satisfactory in his own experience, care being taken to reduce inflammation of the pulp before making the application.

He cautions against the use of arsenic in temporary teeth, preferring repeated applications of carbolic acid. He concludes that, as a substitute for arsenic, cocaine will yet rank number one for pulp devitalization, preferably removing only the bulbous portion of the pulp, leaving the small fibers to continue the nourishment of the tooth.

### ANTISEPTICS—FORMALIN.

At the meeting of the Academy of Stomatology (Phila.),‡ Drs. Kirk and Louis Jack spoke favorably of a dilute solution of formalin (five to ten per cent.), in controlling putrescence, enabling to close the tooth at once.

* Dental Digest, February, 1895.
† Dental Review, February 15, 1895.
‡ International Dental Journal, March, 1895.

DR. McQUILLEN has uniform success in the use of *sodium potassium*, never having had to use it a second time, after once thoroughly burning out or saponifying with it the putrescent matter in the root-canals.

COAGULANTS AND NON-COAGULANTS.

In reply to the paper on "Coagulants," by Dr. E. C. Kirk (*Cosmos*, April, 1894), DR. A. W. HARLAN read a paper bearing the above title before the union meeting of the First and Second District Dental Society of New York,* in which he restates very fully his reasons for believing that coagulants are impenetrable by antiseptics. He stated that the paper was designed to refute the erroneous impressions left last year by Dr. Kirk's paper, its teachings being so opposed to the facts of pure science that it became his public duty to present the present paper. In the discussion which followed the reading, DR. WM. JARVIE said that the result of hearing the papers mentioned, together with that of Dr. Truman on the same subject, was a state of doubt and uncertainty as to what is the best agent, or class of agents, to use in the treatment of pulpless teeth and teeth with putrescent pulps.

It is a case in which the doctors disagree, and yet patients get well by the use of exactly opposite agents.

DR. J. MORGAN HOWE emphasized the value of clinical observation, and thinks that when experimental work contradicts experience, it is the experiments that are likely to be at fault.

PROF. HEITZMAN said that the verdict of the microscope was not in favor of the essayist. When the pulp is destroyed the tenants of the canalicule are killed also, their source of life and nutrition being cut off; the shrunken, dry threads within the canals furnish no pabulum for these gourmands.

DR. S. G. PERRY spoke from the clinical standpoint, being satisfied with the actual results obtained, though not understanding, from the chemical standpoint, what the action of the agent is or why the results follow. The thing for the dentist is "to get there," whether from a theoretical standpoint or not.

---

* Dental Cosmos, March, 1895.

The discussion was carried on to great length, the only conclusion reached being—as put by Dr. I. E. Hill—"we will go right on with what we have been doing for the past thirty or forty years, and have done successfully."

Editorially, Dr. Kirk regrets that the main point at issue—the penetrability of coagulants by antiseptics—was lost sight of in the discussion, in the consideration of collateral and subsidiary problems, which, though of the greatest practical value, are quite aside from the fundamental factor, which should be solidly established upon a basis of truth before the related questions are taken up for investigation and settlement.

### ROOT-CANAL FILLING—SALOL.

Drs. Burchard, Kirk, McQuillen, Register, and others, in the Philadelphia Academy of Stomatology,* testify to uniform success in the use of salol as a root-canal filling material.

Dr. Burchard first uses a solution of sodium peroxide to saponify fatty material, dissolving and driving out the contents of the tubuli. This is neutralized by a weak solution of sulphuric acid, followed by thorough drying with alcohol and hot blast. A portion of salol crystals is taken up with a pair of long-pointed dressing pliers and held above a small flame until it becomes fluid. The closed points are then placed as high up the canal as possible, and slowly opened, the fluid running up the dry canal. An iridium broach is warmed and used to pump the melted salol to the apex, and a point of metal or gutta-percha thrust into the still fluid material.

Dr. Kirk said he had yet to see the first case of apical pericementitis following the use of salol in root-canals, though using it indiscriminately in "immediate" cases and those of recent devitalization. In reply to a question, Dr. Kirk said he did not think he would hesitate to use it in case of wide-open foramen, and if there was any risk of forcing any filling material through he would rather it should be salol than anything else he knew of, "but that was an accident to be guarded against."

*International Dental Journal, March, 1895.

DRS. GUILFORD and KIRK use it in filling the canals of teeth to be implanted, introducing it through the slightly enlarged apical foramen.

DR. H. C. REGISTER uses salol in connection with oxide of zinc, mixing the crystals of salol with the oxide before combining with the liquid. He also uses it by filling the canal and part of the pulp chamber with dry crystals packed down as tightly as possible, and then applying a small nerve instrument warmed slightly. As soon as it touches the salol it liquefies, when it will pass into the most infinitesimal space. Almost as soon as the instrument is withdrawn the salol solidifies into a hard crystalline mass. In putrescent cases, after desiccating thoroughly with hot air, he melts salol into the canals and fills the tooth at once, and has not had any subsequent trouble with such cases.

### THE DENTAL TUBULI.

DR. D. M. CATTELL presented a series of lantern views of tooth-outline before the Northern Illinois Dental Society,* drawing the following, among other lessons from the views :

First—the impossibility of reaching the apical end of all roots; second—the importance of thorough sterilization for the removal of the noxious gases in the tubuli, as well as the pulp canal proper; otherwise, they may penetrate the cementum through the lacunæ and canaliculi, and, reaching the peridental membrane, cause serious trouble. Volatile tonics, capable of passing through the walls between the canal and membrane—such as oil of cassia, or Black's "1, 2, 3," are indicated for this purpose.

### THE TREATMENT OF BICUSPIDS.

A paper on this subject, by Dr. CHARLES W. JENKINS, was read at the meeting of the American Dental Society of Europe.†

Profiting by the suggestions offered very *apropos* by "an invisible and inaudible friend—Jack Tarukappe"(?) who seems to be always on hand at the critical moment, Dr. Jenkins records the following timely hints *soi disant* thus received, to make a

* Dental Review, Feb. 15, 1895.
† Dental Digest, Feb. 1895.

polite excuse to a patient and slip away into the next room and quiet his own tired nerves for five minutes, the faithful friend in the meantime administering invisible chloroform, hypnotizing the patient (!) so that both patients are refreshed by this brief respite; to hold the rubber dam water-tight by means of chloro-percha, without a ligature, thus avoiding the torture of drawing the silk under the gum; in preparing a compound cavity extending far under the gum, rendering the adjustment of the rubber dam very difficult, plug the mouth of the root canal and insert a bit of wood in the center of the crown cavity, leaving sufficient room and retaining shape for emalgam at the neck. Fill that with a good sub-marine amalgam; after it is a little hard, apply the dam as in an ordinary case, withdraw the plug and go ahead.

Intending to follow the prescribed method in preparing an unfortunate bicuspid with two approximal cavities, "Jack" whispered, work for a triumph of common sense, rather than a triumph of art, and instead of making a bicuspid of gold, spotted with natural inlays, preserve the coronal ridge and as much of the denture as possible by filling the crown fissure and the approximal cavities with oxyphosphate, binding together the threatened parts, putting a strong buttress of cement against the coronal ridge, and cover the whole with gold, as thin as consistent with making it secure, the bicuspid will last longer, and look better while it lasts. As "Jack" said: "You can cut an eel from end to end, and cut out and cut off nearly everything that belongs to him and he will swim away pretending to enjoy life the same as ever, but your bicuspid is a creature that gets discouraged when his insides are trifled with!"

PROSETHETICS.

In the New York Odontological Society,* Dr. Hodson asked for suggestions as to how to hold firmly and comfortably in position, a lower partial plate, all of the teeth back of the cuspids being gone, the latter being live teeth and very sensitive, and the ridge a mere thread, very tender and delicate. Among the methods offered, Dr. Jackson advised wedging between the cuspid

*International Dental Journal, March, 1895.

and lateral on each side to obtain a slight space, and extending spring clasps from the plate, fitted to the distal side of the cuspids and extending somewhat towards the front. This, he said, will retain the plate firmly.

At the Union Convention in Buffalo,* Dr. J. B. Wilmott read a paper entitled: "Partial Artificial Dentures a Menace to the Natural Teeth." He observes a marked tendency to the development of caries on approximal and lingual surfaces of teeth in contact with artificial dentures, which he attributes to interference with the free movement of interstitial moisture. Accepting the theory that dental caries is due to the action of acid ptomaines of certain bacteria under favoring conditions, these conditions are all met in the mouth, wearing a partial plate, which is "a perfect fit" and removed but once perhaps in twenty-four hours. The remedy suggested is the use of thin metallic bases, leaving space for free wash over the surfaces of the natural teeth, depending for retention a skillful clasping, or on suction. A cut-off plate, exposing in the median line from three to five-eighths of an inch of the mucous covering of the palatal surface of the mouth, extending across the palate and retained by suction, offers great advantages toward immunity from caries of the palatal and approximal surfaces of the anterior teeth, and also aids in the function of taste, allowing the tip of the tongue to come in contact with the mucous membrane of the palate. Patients should be instructed to remove plates and rinse the mouth, forcing fluid over all the surfaces of the teeth several times a day, and under no circumstances to wear the plate at night.

Dr. S. B. Palmer recommends that the clasps of a partial plate should always have a lug extending over masticating surface of the tooth, or be in the form of a partial cap, relieving pressure on the gum tissues and preventing elongation of the teeth.

Dr. Magill said the clasp should embrace the crown and not the neck of the tooth. He described his method of inserting a screw in a tooth to hold the clasp in place, the clasp fitting over the screw.

* Dental Cosmos, March, 1895.

### PORCELAIN-FACED BICUSPID CROWN.

Dr. A. W. M'Candless, in a paper read before the Northern Illinois Dental Society*, gave his method of making a porcelain-faced bicuspid crown.

The root having been prepared, a band of 22 karat gold, 28 gauge, is fitted as though for an all gold crown, and the buccal portion cut away. The cusp is then tacked to the ferrule at the point farthest from the porcelain front and the gold of the cusp cut down approximating the shape of the porcelain face at that point.

Grind the facing to proper shape, making allowance for the backing, bevel the edges all the way around and back with pure gold, 30 gauge. Catch the backing to the ferrule and cast with sticking-wax, slip off the porcelain and invest in asbestos fiber, exposing only sufficient to unite the backing to the ferrule. Remove the investment, fill the joints where solder is to flow with wax, fill the interior of the crown with plumbago to prevent inflow of solder, complete the soldering of the joint and contour the finished crown, filling the cusp with 14 karat solder. When all is filed or ground to proper shape and finish, force the porcelain to place. (A thin film of cement between porcelain and backing and bending the pins outward when the cement has set, will give sufficient strength to the facing.) A final plating of pure gold gives a beautiful finish which will never tarnish in the mouth.

### TUBE TEETH IN BRIDGE WORK,

Dr. J. H. Spaulding, Paris, contributed to the proceedings of the American Dental Society of Europe† a paper on the use of tube teeth in bridge work.

By his method the natural roots requiring crowns are fitted with cap and pin as usual, the pins being of platinum wire of size to fit the tube of the teeth. The tube teeth are ground to fit the roots in circumference, and with cups and pins in places an impression is taken and model made. Each tube-tooth is con-

* Dental Review, February 15, 1895.
† Dental Digest, February, 1895.

caved on the under side and a thin plate of pure gold burnished to the ground surface, in which a hole is pierced to receive the pin and the concavity filled with 20 k. solder. Each crown of attachment is then placed in position on the cap and pin and held with wax, and all removed together; the porcelain is then slipped off the pin and the cap and base united by the wax invested for soldering with 20 k. solder after the wax is boiled out. The teeth which bridge a space are so ground as to leave a self-cleaning space before receiving the gold base as before described.

All the teeth being thus prepared with gold bases and pins, with caps and base united for the prepared roots, they are all put in position and adjusted as desired on the model and waxed together. The bridge is then lifted off the model, the porcelains removed and the bridge invested for final soldering.

Thus, none of the porcelain teeth are left in place at any time while soldering, but little gold or solder is used, and almost none presented to view when the bridge is in place. As the final step the tube teeth are secured to the pins by means of melted sulphur.

The same method is used in crowning bicuspids.

Let the prejudice against these English tube teeth—because of their unnatural shapes—be overcome and a demand created for them, and the demand will force their manufacture in more esthetic molds. They are the strongest teeth made; they can be ground and polished without injuring their appearance; they are easily adjusted; repair is very easy, though seldom required.

### ORTHODONTIA—JUMPING THE BITE.

DR. C. S. CASE,* in a reply to Dr. Ottolengui's "reply" to a previous article by Dr. Case on this subject, maintains his position that, while there is nothing in the natural construction of the tempero-maxillary joint, or in the character of the inter-articular tissues, to prevent the jaw from being permanently carried forward sufficiently to "jump the bite," or to interfere with the ultimate possibilities of perfect dental occlusion, yet, that in more than half the cases of abnormal protruding upper

* Dental Review, February 15, 1895.

jaw, this is not the remedy for the deformity. A careful preliminary study of the face, not merely of the teeth, but of the entire face, and especially of the relative position of the lower jaw and chin to the forehead, bridge of the nose, etc., may show that to protrude the lower jaw by "jumping the bite" would increase the deformity rather than remedy it. The upper jaw is as often too large in its relation to the other bones of the face as the lower is too small or receding. When this is the case reduction of the upper anterior protrusion is indicated—usually accomplished by the extraction of two upper bicuspids and forcing back the anterior portion of the bones, carrying back the six anterior teeth which gave abnormal prominence to the middle features.

Dr. Case also repeats his criticism of the case cited by Dr. Ottolengui, who claims that Dr. Kingsley permanently corrected the deformity by jumping the bite "in two weeks." Dr. Case maintains that the necessary structural changes at the tempero-maxillary joint being impossible of accomplishment in that space of time, though the models presented apparently showed the results claimed. There is nothing to prevent placing the lower jaw forward the full width of a tooth and taking an impression of the face with the jaws in that position, but to secure permanent results something more is necessay than the mere temporary relative change in position. Nothing could have occurred at the joint, the real seat of requisite surgical change, to give the right to claim a permanent correction of the deformity.

[*To be Continued.*]

